# Effects of Physical Activity and COVID-19 on Healthy Student Strengths in the University System: Implications for Post-Pandemic Management

**DOI:** 10.3390/ejihpe14010016

**Published:** 2024-01-19

**Authors:** Ramón Gómez Chacón, Jose Manuel Nuñez Sánchez, Pablo Gálvez Ruiz

**Affiliations:** 1 Cardenal Spínola CEU University Studies Center, 41930 Bormujos, Spain; 2 Faculty of Economics and Management, Universidad de Málaga, 29013 Málaga, Spain; josemanuel.nunez@uma.es; 3 Faculty of Law and Social Sciences, Valencian International University, 46002 Valencia, Spain; pgalvez@universidadviu.com

**Keywords:** healthy student strengths, physical activity, mental health, COVID-19, professor’s guidance

## Abstract

The COVID-19 pandemic has profoundly affected the physical, mental, and social well-being of millions worldwide. It has also brought about abrupt disruptions to the entire university system, whose students form a crucial segment of society. The pandemic’s effects on student education and well-being have been particularly significant. One of the primary consequences has been a drastic reduction in physical activity levels among students, leading to mental and physical health problems. Despite the rapid growth in the literature exploring student experiences during the pandemic, there is a paucity of research on how this decline in physical activity has affected the five strengths of the healthy student: optimism, self-efficacy, resilience, engagement, and hope. Therefore, the aim of this investigation is to examine the relationship between physical activity levels and the five strengths of the healthy student at two different time points (pre-COVID-19 and COVID-19) through the International Physical Activity Questionnaire (IPAQ) and the Healthy Student Questionnaire. The study involved 897 participants, with 290 participating in the pre-COVID-19 phase and 607 participating in the COVID-19 phase. The results revealed significant differences in the five strengths between the two periods. Students who engaged in physical activity exhibited significantly higher optimism scores in the pre-COVID-19 phase. During the COVID-19 phase, physically active students demonstrated significantly higher scores in optimism, resilience, and self-efficacy. These findings provide clear guidance for university administrators seeking to enhance student well-being in a post-pandemic world and in the face of future disruptions. Universities should consider implementing physical exercise programs for their students to promote psychosocial well-being and provide training and resources to equip faculty members with new skills to better understand and support students’ perceptions.

## 1. Introduction

The pandemic caused by the COVID-19 outbreak posed significant challenges for the entire educational community. Specifically, in the university context, changes to remote teaching and methods based on new technologies produced significant interruptions in teaching and learning [1], thus adding to the difficulty that many students have in adapting to the teaching/learning environment of the university [2].

Parallel to this situation, the restrictions caused by the pandemic provoked a profound change in the lifestyle of university students with additional negative consequences such as reduced physical activity (PA) [3,4], decreased well-being [5] and psychological well-being [6] and a worsening of mental health [7]. University students are considered a population vulnerable to psychological disorders, and additional academic stressors such as anxiety and insecurity can lead to students having poor academic performance [8].

On the other hand, the healthy student concept was defined by Gómez-Chacón [9] and is based on the healthy employee model of Salanova et al. [10]. It is founded on the concept of psychological capital, which is defined as a positive psychological state of individual development. These individuals have psychosocial strengths such as resilience, self-efficacy, optimism, hope and engagement [10].

In light of the unprecedented circumstances brought about by the pandemic, this study delves into the impact it has had on university students particularly with regard to the five strengths of the healthy student. Echoing the call of Núñez-Sánchez et al. [11], this research endeavors to provide actionable insights that can empower organizations to effectively address the formidable challenges posed by COVID-19. Additionally, it aims to equip university institutions with the knowledge and tools necessary to navigate similar crises in the future, drawing upon the lessons learned during this demanding period.

### 1.1. Literature Review

#### 1.1.1. Physical Activity Levels in COVID-19 Times

Regular PA is an essential element of a healthy lifestyle. The World Health Organization [12] recommends that in order to obtain significant benefits for their health, adults should accumulate between 150 and 300 min of moderately intense aerobic exercise each week or at least between 75 and 150 min of vigorously intense aerobic exercise. Alternatively, they should achieve an equivalent combination of moderately and vigorously intense aerobic exercise. In addition, the daily required step count for adults is estimated by various studies to be between 4000 and 18,000 [13,14]. However, the student population reduced their amount of PA during the coronavirus crisis [3], and some studies [15,16] warn of the negative consequences for the physical and mental health of these reductions, as well as their impact on the quality of life.

In addition to the confinements, the situation was aggravated by restrictions on sports facilities and a lower availability of sports services due to social distancing, which prevented university students from maintaining their frequency of PA and regular practice. In this context, motivation in the practice of physical activity is of vital importance, and exercise programs are motivating for students [17]. Additionally, the confinement situation prevented students from reaching the sleep levels recommended for their psycho-physical health. Therefore, they were at risk due to both inactivity and reduced sleep [16].

In this context, Kirschner et al. [6] found a positive association between PA and mental well-being in university students, while the decrease in PA during the pandemic, compared with the previous period was associated with greater depressive symptoms [18]. Therefore, considering that regular PA is positively related to enthusiasm and psychological well-being [19], and that well-being is especially relevant for students as it influences their academic and professional development [20], regular PA could serve as a protective factor for the mental health and educational achievement of students during the COVID-19 restrictions [7].

On another note, academic stress, which is defined as a physiological, emotional, cognitive and behavioral activation reaction to academic stimuli and events [21], is known to impact very significantly on university students. These impacts were greater during the COVID-19 pandemic when higher levels of stress were present compared with the pre-pandemic period [22,23,24]. Organized physical exercise has positive psychological effects for the individual, preventing stress and increasing self-control and self-reliance as well as allowing time to avoid unpleasant thoughts, emotions and behaviors [25,26]. Conversely, it has been reported that a significant decrease in PA leads to a worse quality of life, insomnia, increased depression, psychiatric disorders, anxiety, and mood states [27,28,29,30].

#### 1.1.2. Healthy Students’ Strengths during COVID-19

The concept of the healthy student was introduced by Gómez-Chacón et al. [9] and describes a positive psychological state of individual development characterized by five distinct strengths according to Luthans and Youssef-Morgan [31]: having confidence, making a positive attribution (optimism or positive emotions) about success now and in the future, persevering toward goals and, when necessary, redirecting paths to goals (hope or competence) to succeed, and when embraced by problems and adversity, sustaining and rebounding and even beyond (resilience) to achieve success. Finally, there is the engagement strength, defined by Schaufeli et al. [32] as the positive affective state, relatively persistent, characterized by vigor, dedication and absorption or concentration. These strengths have a positive impact on student psychological well-being. However, different studies show a psychological negative impact of the COVID-19 emergency on college students [33].

Firstly, hope and optimism act as cognitive factors in protecting and helping to reduce life stressors in students’ lives, being considered to be fundamental in adapting to horrendous life events by trusting in a greater future, and they may act as interceding factors [34]. Therefore, the more optimistic an individual is, the lower the depression level and the higher life satisfaction [35]. Following Jiang et al. [36], university students who express positive emotional experiences will develop positive and optimistic attitudes. Even if they encounter negative life events, they are more likely to adopt positive coping methods to deal with problems, enhance the acceptability of negative events, and thus improve the level of life satisfaction.

Distress during the pandemic was so severe in certain students that they felt hopeless [37], and hope plays a crucial role as a coping mechanism in maintaining university student´s life satisfaction [35]. These authors stated that hope could very much be vital for university undergraduates and aid them in overseeing concerns during pandemics, and having a feeling of hope safeguards the well-being of university undergraduates, reducing the pessimistic impacts of adverse conditions, as demonstrated by many investigations [34].

Second, in these COVID-19 times, resilience was found to reduce the negative effects of pandemic-associated stress on the life satisfaction and psychological well-being of students [38]. In this regard, it is important to note that university students are considered as a risk group in terms of psychological resilience; a significant difference between psychological resilience and anxiety levels was found in favor of students who had higher PA levels [39]. This pandemic situation resulted in decreased resilience even more in students who did not engage in PA [40]. When an individual is equipped with adequate resilience, they are less likely to suffer from stress, anxiety, loneliness, depression and post-traumatic stress [41]. In other words, resilience presents a potential role in safeguarding the psychological health and well-being of students during the coronavirus outbreak [38].

Thirdly, student´s engagement is considered a key success element. The student engagement construct is related to positive psychology [42], focusing on those factors that engage students either with education or with PA [43]. Student engagement with education and/or PA is a key factor in enabling students to overcome the negative consequences that the pandemic has had on them. Research carried out during the pandemic states that students’ well-being and learning are entangled with an engagement with the university, with seeing the worth, purpose, and recognition for what they do and the importance of emplaced learning to do so [44]. These authors found there was a silent means of disengagement, less quantifiable than dropout rates, but that constitutes a qualitative issue that is pressing to address. In comparison to the pre-lockdown period, students feel less productive and less engaged, which affects their emotional well-being and their engagement [45].

Fourthly, the strength of self-efficacy is analyzed in the academic context, where students with high self-efficacy tend to show more healthy behaviors, which favors the development of healthy life habits [46]. Furthermore, the stronger the sense of self-efficacy, the healthier behaviors and the more significant the positive emotion expressed [47].

Finally, the authors considered it opportune to explore how the students had felt guided by their professors, as the scientific literature shows that online classes may have psychological effects on university students due to continuous isolation and lack of interaction with fellow students and teachers [48]. In addition, Cunha et al. [45] showed students’ troubles during the pandemic, underlining that student empathy seems to be a quick and easy initiative to improve students’ performance and mood. Indeed, empathy is critical for cultivating good student–teacher relationships, which is positively related to student achievement [49]. On the other hand, student perceptions on the ill preparedness and disengaging responses of educators emerged as another factor that affected student experience [50]. Moreover, perceived lack of support from educators and reduced interactions with peers further increased stress among students [51]. Cameron and Rideout [52] confirmed the relevancy of support, especially for first-year students, while Fang et al. [53] stated that engaging and caring lecturers who exercise flexibility in their teaching could provide the platform and boost motivation as a supportive intervention for students. Therefore, instructors should ensure that their attitudes are positively perceived by students to facilitate improved perceived learning outcomes [54].

There is little scientific literature that relates physical activity to healthy student variables, although it is well known how physical activity impacts on these variables in the work environment [55] as well as in other areas [56,57]. However, it has been shown that more resilient students cope successfully with stressful times, such as studying abroad, adapting to a new university, or adopting in a different language [58]. Furthermore, students with high levels of resilience are significantly associated with lower levels of stress [58]. The main objective of this research was to investigate the impact of the pandemic on university students, specifically on the five strengths of the healthy student, and the influence of PA and professor´s guidance on these five strengths. There are three objectives of this study:

− To analyze the relationship between the practice of PA and each of the five strengths of the healthy student at two very different moments, pre-COVID (January 2020) and COVID (January 2022).
− To find out how COVID affected the five strengths of the healthy student.
− To study the influence of student’s perception of their lecturers´ guidance on each of the five strengths of the healthy student in times of COVID.

## 2. Methods

### 2.1. Procedure and Participants

After reviewing the literature on the impact of PA on different academic variables and contexts, the relationship between PA and the healthy student was considered, including the COVID-19 influence. In this sense, a pre-COVID survey was used including 2 validated instruments: IPAQ [59] and the Healthy Student Questionnaire [9].

The study has been carried out with a sample of university students in Spain, using an online questionnaire (Google form) for data collection at two specific moments: pre-COVID with data collection between January 11 and February 7, 2020, and COVID, where data collection began on January 20, 2022 and ended on February 21, 2022. The reason for closing the questionnaire in February is due to the completion of the first semester in the Spanish university system. All the participants were informed of the voluntary nature, anonymity and confidentiality of their participation, obtaining the informed consent from all of them. The information was collected using a convenience sampling strategy, with the total number of participants being 897: 290 in pre-COVID and 607 in the COVID period, including students who met the criteria for enrollment at the university and were over 18 years old, and excluding high-level or competitive athletes. The specific characteristics of the sample in relation to gender and level of PA are found in Table 1.

### 2.2. Measurements

This study has as its starting point the Health and Resilient Organization tool (HERO) [10], using for the present investigation the questionnaire of healthy employees in the validated version adapted to the educational context [9], which is made up of 5 dimensions and a total of 40 items: optimism (6 items), engagement (structured in the sub-dimensions vigor, dedication, and absorption) (18 items), resilience (7 items), hope (composed of the mental and emotional sub-dimensions) (6 items), and self-efficacy (3 items). Responses were provided using a seven-point Likert scale ranging from 0 (never) to 6 (always).

An analysis was carried out to test the goodness of fit of the measurement model in the two study samples as well as an analysis of internal consistency through Cronbach’s alpha indicator. The model fit indexes were good in both models (Table 2), being within the range established in the specialized literature. Regarding internal consistency, the analysis showed that all the measured constructs were reliable with Cronbach’s a values ranging between 0.77 and 0.89 (moment 1: pre-COVID) and between 0.75 and 0.90 (moment 2: COVID).

## 3. Results

In a first analysis (Table 3), the descriptive statistics (i.e., means, standard deviations, univariate normality using skewness and kurtosis, and Kolmogorov–Smirnov test) of the strengths of the healthy student were checked, obtaining higher mean values in pre-COVID for the five dimensions. The self-efficacy dimension did not fit a normal distribution at moments 1 and 2, nor did the hope dimension at moment 2. In addition, the correlations between the different dimensions at the two moments were analyzed (pre-COVID /COVID), obtaining significance and correlations between moderate and high [60].

In a second step, the authors examined whether the strengths of the healthy student showed significant differences depending on the two moments and gender at each moment (Table 4), using the non-parametric Mann–Whitney U-test. The results showed significant differences in the five strengths between moments 1 (pre-COVID) and 2 (COVID). Taking gender into account, the male sample in both pre-COVID and COVID showed higher scores. Resilience strength obtained significant differences in the two moments and optimism only in moment 2 (COVID).

Table 5 shows the results between the practice of PA and the different strengths of the healthy student at the two moments of the study, using the ANOVA test for analysis. Moment 1 (pre-COVID) shows significant differences in optimism between the students practicing high PA (M = 5.12) and average (M = 4.86) compared to students who performed little or no PA (M = 4.26). However, the other four strengths do not present significant differences between the different groups of PA levels.

On the other hand, at moment 2 (COVID), there are significant differences in optimism between students practicing high (M = 3.00) and low (M = 2.61) PA. At the same time, the resilience strength presents significant differences between high PA (M = 3.98) and medium PA (M = 3.73) and low PA (M = 3.59). In addition, the self-efficacy strength presents significant differences between high PA (M = 4.14) and low PA (M = 3.76). Meanwhile, the engagement and competition do not display significant differences between the different groups of PA practice.

As a novelty in the questionnaire used at moment 2 (COVID), the student´s guidance perception question was added to find out how they had felt guided by the professors in times of COVID, and how this perception impacts the strengths of the healthy student. In this sense, when performing an ANOVA analysis between the strengths of the healthy student and the perception of being guided by the teacher (Table 6), the results showed that the students who responded feeling very well oriented showed significant differences in all the strengths of the healthy student with respect to the rest of the responses, pointing out that the better guided the students felt, the better the indicators of the strengths of the healthy student show the results.

## 4. Discussion and Conclusions

The main objective of this research was to investigate the impact of the pandemic on university students, specifically on the five strengths of the healthy student, and the influence of PA and professor´s guidance on these five strengths. This global target has been divided into three specific research objectives that will be discussed in this section. Based on what has been set out, the authors come up with possible proposals for university management in the post-pandemic era. Researchers have not found similar studies in the literature, so this investigation could fill this gap.

The first objective of this study was to analyze the relationship between the practice of PA and each of the five strengths of the healthy student at two very different moments, pre-COVID (January 2020) and COVID (January 2022). At the pre-COVID time, students who engaged in both high and medium PA scored significantly higher on optimism than students who engaged in low or no PA. As for the COVID-19 moment, students who engaged in high PA scored significantly higher on optimism and self-efficacy than students who presented low or no PA; results aligned with Aguirre-Loaiza et al. [61]. On the other hand, students who engaged in high PA had significantly higher resilience scores than students who engaged in medium and low or no PA, which was in line with Olmos-Gomez [40], who stated that this decreased resilience was one of the main problems in students who did not engage in physical activity, with resilience being a very important factor in protecting the mental, emotional and psychological health of an individual [62]. Likewise, lower PA was associated with reduced engagement, presenting other different negative outcomes for students, such as motivation.

This direct relationship between PA and student strengths is in agreement with Lukács [15] and Luciano et al. [16], as students reported lower levels of PA and psychological well-being during the pandemic. Since students are vulnerable to psychological distress, and regular PA can reduce symptoms of this, it is vital to study how psychological well-being, perceived health status and PA among university students alter during the coronavirus pandemic [15]. It is possible to conclude that PA plays a fundamental role, which aligns with Liu et al. [46] who highlighted the importance of physical health and emotional support. In other words, maintaining or enhancing regular PA during stressful life events such as the COVID-19 pandemic is indispensable [35]. According to Ren et al. [63], the higher the PA of university students, the better their mental health status, interpersonal relationship status and emotional status. On the contrary, the less PA university students had, the unhealthier their mental state. The importance of PA for students is demonstrated, even more so in times of a pandemic, because the greater the practice of PA, the better results in the five strengths of the healthy student.

These findings show the importance for students of engaging in PA to combat the negative effects on student strengths, such as optimism, resilience, self-efficacy, hope and engagement, in times of a pandemic. Therefore, the results obtained are in line with Granero-Jiménez et al. [64] who showed significantly higher scores on psychological well-being for those with a high level of PA compared to those with a lower level of activity. Nevertheless, following Wilczyńska et al. [65], it should be noted that the increased level of COVID-19 anxiety also affected the students’ motivation for PA.

The second research objective was to find out how COVID affected the five strengths of the healthy student, observing significant reductions in all the strengths. These conclusions are aligned with the emerging literature on the negative impacts of the pandemic on students’ well-being—emotional and/or psychological—which are effects found worldwide [15]. In other words, the pandemic has negatively affected psychosocial aspects in students, which is in agreement with the study by Yao et al. [66], who warned that university students are likely to be more susceptible to the negative impact of the pandemic as they previously had reduced psychological well-being, having a significant (mainly negative) impact on both the overall learning experience of our university students and their psychological well-being [1]. Specifically, this decrease in positive emotions, such as hope, is in line with Lee [37] who noted that during COVID, certain students felt hopeless. For reduction in engagement, the results obtained are related to the study of Daniels et al. [67], while resilience is in line with the study of Cunha et al. [45]. However, Serrano-Sarmiento et al. [68] found generally high levels of resilience among their sample of university students in confinement. Finally, the reduction in self-efficacy is in line with the study of Lin [69].

The third research objective sought to investigate the influence of students’ perceived guidance from their lecturers on each of the five strengths of the healthy student during the COVID-19 pandemic. Notably, students who reported feeling well guided by their lecturers demonstrated significant improvements in optimism, resilience, self-efficacy, hope, and engagement. In other words, students who perceived stronger guidance from their teachers exhibited higher scores in the five strengths. These results are in line with Cameron and Rideout [52] who confirmed the relevancy of professor support, while Fang et al. [53] stated that engaging and caring lecturers who exercise flexibility in their teaching could provide the platform and boost motivation as a supportive intervention for students. Emotional support is vital for student well-being and continued learning [1]. The importance for students to feel well guided by their professors in times of a pandemic is noteworthy, which is in line with Cameron and Rideout [52].

### 4.1. Conclusions

The conclusions of this study can be summarized as follows. (1) The five strengths of the healthy employee show significant differences between the pre-COVID and COVID moments, obtaining lower scores in COVID. (2) In the pre-COVID moment, students who practiced PA, both high and medium, presented significantly higher scores in optimism than students with low or no PA. (3) During COVID, students who engaged in high PA had significantly higher scores on optimism and self-efficacy than students with low or no PA. In addition, students who engaged in high PA had significantly higher scores in resilience than students who engaged in medium and low or no PA. Therefore, it seems advisable to explore opportunities for improving PA levels of students by enhancing or developing sports facilities and infrastructure at universities in order to facilitate and encourage students to engage in PA in the post-pandemic era.

Conversely, during the COVID-19 period, students who reported feeling well supported by their professors demonstrated significant improvements in optimism, resilience, self-efficacy, hope, and engagement. This suggests that the strength of the student–teacher relationship positively influences the development of the five strengths of the healthy student.

### 4.2. Limitations and Future Lines

The present research offers interesting results for the post-pandemic period. However, it is not without some limitations, such as the need for even larger samples, segmentation by locations, faculties, academic courses, or gender. First, once the importance of professor´s guidance has been observed, it would be necessary to broaden the factors that influence students’ perception of it by means of reliable and validated instruments. Second, it has not been analyzed which of these universities offer well-being programs, so we think it would be necessary in future studies to differentiate between universities that have implemented well-being programs for their students and universities that have not. Third, PA has been measured through a validated questionnaire but, if possible, it would be advisable to also use objective measurements. Fourth, although the sample size is enough for this research, it could include more sociodemographic characteristics. Fifth, despite the study having an adequate sample size, the absence of some demographic data prevents the performance of certain analyses that would allow more segmented results to be obtained.

On the other hand, it would also be of interest to complement the quantitative methodology with a qualitative one, featuring in-depth interviews with students and university managers. Therefore, it is a good idea to continue this type of research in the future in order to help universities take better care of students’ physical and mental health by maintaining and even increasing their PA levels and the five strengths of the healthy learner so important for university students in their learning and well-being.

Finally, this research has not analyzed the motives for participation in PA, which could help promote motives that increase sports participation. This should be analyzed in future research.

### 4.3. Managerial Perspectives

This study has yielded a series of recommendations for enhancing educational management practices. The foremost recommendation is for universities to prioritize the well-being and health of their students, proactively ensuring that their study experience remains unaffected by unforeseen circumstances, such as those encountered during the pandemic. This is in line with Dood et al. [1], who asserted that supporting the health, well-being, and learning experiences of all students should be a high priority now and in post-pandemic times.

The importance of PA has been demonstrated, especially in times of a pandemic and online classes, for maintaining good physical and mental health, while keeping up the five strengths of the healthy student. For this reason, the second recommendation is the inclusion of sports classes at university, linking them to a subject of the degree studied. This is in line with Ren et al. [63], who stated that universities should implement PA education. Due to the importance of PA, students should be encouraged by positive psychological interventions [45], stimulated to exercise more [70], and motivated to participate regularly in physical activities to improve physical fitness [63]. In this regard, it would also be important to create and facilitate attractive and interesting environments for students to practice PA. Universities should also stimulate students to actively participate in campus activities by providing a healthy environment for them [35].

In terms of the five strengths of the healthy student, universities should develop action plans, especially in the post-pandemic period, to maintain or recover pre-pandemic levels. Along these lines, universities should develop strategies to meet the specific needs of students by promoting psychological resilience [48]. Moreover, students should be trained on how to use the power of hope when they face an environmental crisis or have trouble maintaining life satisfaction [35]. Providing hope-related interventions to university students are even recommend to cope with challenges and improve life satisfaction [35], and students’ emotional well-being in difficult times [45]. Other researchers also advocate exercise intervention for stay-at-home students to improve their emotional control in order to alleviate their anxiety and depression in the face of unexpected events [71].

Finally, the importance of lecturers’ support and guidance in such difficult times has been demonstrated. This is why university staff, especially professors, should show more empathy toward students’ struggles [49]. Universities can increase the number and support of counsellors and social supports by ensuring that students are aware of existing support systems and that these are accessible to all students [72]. In this sense, it would be necessary to support and train professors at the university level, to equip them with the knowledge and tools necessary to achieve the goal of making students feel well- guided and accompanied in times of a pandemic and post-pandemic. For this reason, universities should provide professors with professional training, support and adequate resources [53].

## Figures and Tables

**Table 1:** Characteristics of the sample.

Variable	Pre-COVID	COVID
	N/%	N/%
Gender		
Male	150/51.7	259/42.7
Female	140/48.3	347/57.2
Lost		1/0.1
Type of PA		
Low	47/16.2	173/28.5
Moderate	82/28.3	303/49.9
High	161/55.5	131/21.6

**Table 2:** Goodness of fit indexes for models 1 (pre-COVID) and 2 (COVID).

Moment	χ²/gL	CFI	IFI	TLI	PCFI	RMSEA
1: Pre-COVID	1.87	0.910	0.911	0.901	0.827	0.055 (CI = 0.051, 0.060)
2: COVID	2.32	0.941	0.941	0.934	0.837	0.047 (CI = 0.044, 0.050)

**Table 3:** Descriptive statistics and correlations.

Descriptive	Moment 1: Pre-COVID			Moment 2: COVID		
	Mean ± SD	Sk/Ku	K − S	Mean ± SD	Sk/Ku	K − S
OPTIMSM	3.91 ± 1.07	−0.43/−0.57	0.15	2.95 ± 1.23	−0.29/−0.29	0.13
ENGAG.	3.88 ± 0.97	−0.38/0.28	0.50	3.21 ± 1.05	−0.17/0.05	0.31
RESILIE.	4.05 ± 1.10	−0.52/0.23	0.12	3.77 ± 1.03	−0.37/0.14	0.07
HOPE	4.09 ± 1.00	−0.34/−0.15	0.07	3.76 ± 1.03	−0.48/0.59	0.01 ^1^
SELF-EFF.	4.30 ± 1.07	−0.40/−0.23	0.02 ^1^	3.97 ± 1.16	0.52/5.53	0.00 ^2^
Correlations	OPTIMISM	ENGAG.	RESILIE.	HOPE	SELF − EFFI.	
OPTIMSM	1					
ENGAG.	0.64 ^2^/0.56 ^2^	1				
RESILIE.	0.52 ^2^/0.51 ^2^	0.72 ^2^/0.68 ^2^	1			
HOPE	0.55 ^2^/0.34 ^2^	0.70 ^2^/0.52 ^2^	0.69 ^2^/0.57 ^2^	1		
SELF-EFF.	0.47 ^2^/0.34 ^2^	0.59 ^2^/0.51 ^2^	0.59 ^2^/0.56 ^2^	0.69 ^2^/0.63 ^2^	1	

Note. SD = standard deviation, Sk/Ku = skewness/kurtosis, K − S = Kolmogorov−Smirnov, ^1^ = *p* < 0.05, ^2^ = *p* < 0.01.

**Table 4:** Average assessment of the strengths of the healthy student.

	OPTIMISM	ENGAG.	RESILIE.	HOPE	SELF-EFFI.
Total sample					
Pre-COVID	3.91 ± 1.07	3.88 ± 0.97	4.05 ± 1.10	4.09 ± 1.00	4.30 ± 1.07
COVID	2.95 ± 1.23 ^2^	3.21 ± 1.05 ^2^	3.77 ± 1.03 ^2^	3.76 ± 1.03 ^2^	3.97 ± 1.16 ^2^
Gender pre-COVID					
Male	4.01 ± 1.02	3.95 ± 0.95	4.21 ± 0.96	4.10 ± 0.96	4.32 ± 1.10
Female	3.80 ± 1.11	3.81 ± 0.99	3.88 ± 1.22 ^2^	4.08 ± 1.05	4.28 ± 1.04
Gender COVID					
Male	3.15 ± 1.18	3.28 ± 1.04	3.88 ± 1.00	3.76 ± 1.01	4.00 ± 1.22
Female	2.79 ± 1.25 ^2^	3.16 ± 1.05	3.69 ± 1.04 ^1^	3.75 ± 1.05	3.95 ± 1.11

Note. ^1^ = *p* < 0.05, ^2^ = *p* < 0.01.

**Table 5:** ANOVA results between PA and the strengths of healthy student in pre-COVID and COVID.

		Moment 1: Pre-COVID		Moment 2: COVID	
Dimensions	TPA	N	Mean ± SD	N	Mean ± SD
OPTIMISM	H	161	5.12 ± 1.02 ^3^	173	3.00 ± 1.15 ^1^
	M	182	4.86 ± 1.08	303	2.81 ± 1.19
	L	47	4.26 ± 0.97	131	2.61 ± 1.28
ENGAGEMENT	H	161	4.95 ± 0.92	173	3.20 ± 1.06
	M	182	4.81 ± 1.06	303	3.22 ± 1.11
	L	47	4.79 ± 0.98	131	3.08 ± 1.09
RESILIENCE	H	161	5.09 ± 1.07	173	3.98 ± 0.97 ^2^
	M	182	5.04 ± 1.10	303	3.73 ± 1.01
	L	47	4.94 ± 1.22	131	3.59 ± 1.10
HOPE	H	161	5.11 ± 0.97	173	3.88 ± 0.96
	M	182	5.08 ± 1.04	303	3.74 ± 1.03
	L	47	5.01 ± 1.03	131	3.63 ± 1.11
SELF-EFFICACY	H	161	5.33 ± 1.03	173	4.14 ± 1.07 ^1^
	M	182	5.31 ± 1.11	303	3.97 ± 1.08
	L	47	5.18 ± 1.14	131	3.76 ± 1.39

Note. TPA = type of PA, H = high, M = moderate, L = low, N = sample, SD = standard deviation, ^1^ = *p* < 0.05, ^2^ = *p* < 0.01, ^3^ = *p* < 0.001.

**Table 6:** Perception of being guided by the teacher and relationship with strengths of the healthy student (moment 2: COVID).

	Perception of Being Guided (Expressed in Mean ± Standard Deviation)					
Dimensions	Very Misguided (*N* = 56)	Misguided (*N* = 109)	Neither Good nor Bad (*N* = 234)	Well Guided (*N* = 156)	Very Well Guided (*N* = 52)	Total (*N* = 607)
OPTIM ^3^	2.21 ± 1.41	2.64 ± 1.12	2.78 ± 1.13	3.04 ± 1.13	3.39 ± 1.34	2.82 ± 1.21
ENG ^3^	2.43 ± 1.13	2.69 ± 0.99	3.14 ± 0.95	3.66 ± 0.94	4.15 ± 1.08	3.21 ± 1.10
RES ^3^	3.23 ± 1.02	3.43 ± 0.93	3.67 ± 0.98	4.12 ± 0.91	4.50 ± 1.07	3.77 ± 1.03
HOPE ^3^	3.11 ± 1.12	3.56 ± 1.07	3.71 ± 0.98	4.02 ± 0.90	4.28 ± 0.96	3.76 ± 1.03
SEFF ^3^	3.44 ± 1.24	3.70 ± 1.10	3.90 ± 1.05	4.17 ± 0.97	4.87 ± 1.57	3.97 ± 1.16

Note. OPTIM= Optimism, ENG = Engagement, RES = Resilience, SEFF = Self-Efficacy, ^3^ = *p* < 0.001.

## Data Availability

Please contact the corresponding author to obtain the data.
